# Genetic Diversity and Molecular Epidemiology of Circulating Respiratory Syncytial Virus in Central Taiwan, 2008–2017

**DOI:** 10.3390/v14010032

**Published:** 2021-12-24

**Authors:** Chun-Yi Lee, Yu-Ping Fang, Li-Chung Wang, Teh-Ying Chou, Hsin-Fu Liu

**Affiliations:** 1 Department of Pediatrics, Chang Bing Show Chwan Memorial Hospital, Changhua 505029, Taiwan; lee821083@gmail.com (C.-Y.L.); y20050401@gmail.com (Y.-P.F.); 2 Institute of Clinical Medicine, National Yang Ming Chiao Tung University, Taipei 112304, Taiwan; tychou@vghtpe.gov.tw; 3 Department of Pathology and Laboratory Medicine, Taichung Veterans General Hospital, Taichung 40705, Taiwan; lcwang@vghtc.gov.tw; 4 Department of Pathology and Laboratory Medicine, Taipei Veterans General Hospital, Taipei 11217, Taiwan; 5 Department of Medical Research, Mackay Memorial Hospital, Taipei 25160, Taiwan; 6 Institute of Bioscience and Biotechnology, National Taiwan Ocean University, Keelung 202301, Taiwan; 7 Institute of Biomedical Sciences, MacKay Medical College, New Taipei City 25245, Taiwan

**Keywords:** respiratory syncytial virus, genotype, G protein, Taiwan

## Abstract

In this study, we investigated the molecular evolution and phylodynamics of respiratory syncytial virus (RSV) over 10 consecutive seasons (2008–2017) and the genetic variability of the RSV genotypes ON1 and BA in central Taiwan. The ectodomain region of the G gene was sequenced for genotyping. The nucleotide and deduced amino acid sequences of the second hypervariable region of the G protein in RSV ON1 and BA were analyzed. A total of 132 RSV-A and 81 RSV-B isolates were obtained. Phylogenetic analysis revealed that the NA1, ON1, and BA9 genotypes were responsible for the RSV epidemics in central Taiwan in the study period. For RSV-A, the NA1 genotype predominated during the 2008–2011 seasons. The ON1 genotype was first detected in 2011 and replaced NA1 after 2012. For RSV-B, the BA9 and BA10 genotypes cocirculated from 2008 to 2010, but the BA9 genotype has predominated since 2012. Amino acid sequence alignments revealed the continuous evolution of the G gene in the ectodomain region. The predicted N-glycosylation sites were relatively conserved in the ON1 (site 237 and 318) and BA9 (site 296 and 310) genotype strains. Our results contribute to the understanding and prediction of the temporal evolution of RSV at the local level.

## 1. Introduction

Human respiratory syncytial virus (RSV) is a leading viral agent of acute lower respiratory tract infections (LRTIs) in young children and vulnerable adults worldwide [1,2]. Children older than 2 years are estimated to have experienced a least one episode of RSV infection, and recurrent RSV infections are common throughout life. Despite the highest incidence of RSV infection being noted in young children, the burden of RSV is comparable to that of influenza throughout the course of life [1]. Although two drugs have been approved to prevent or treat RSV LRTIs in pediatric patients, palivizumab for prophylaxis and aerosolized ribavirin for treatment, these LRTIs contribute to high hospitalization rates, economic costs, and childhood mortalities, particularly in developing countries [2,3].

RSV belongs to the recently defined pneumoviridae family, orthopneumovirus genus, and consists of a single-stranded negative-sense RNA genome packaged in a lipid envelope. The RSV genome is approximately 15.2 kb and contains 10 genes encoding at least 11 proteins. The RSV G protein is a type II transmembrane glycoprotein and is key to RSV attachment and pathogenesis. The G protein contains two hypervariable regions (HVR) spanning a 13 amino acid length centrally conserved cysteine-rich domain. HVR1 is located at amino acids (aa) 164–186 and HVR2 at aa 282–321. The G protein, especially HVR2, is highly genetically diverse and prone to be under selection pressure [4,5]. These genetic and antigenic variabilities in the G protein are widely used for molecular characterization. RSVs have been classified as subgroups A and B (RSV-A and RSV-B) based on genetic analyses and monoclonal antibody neutralization. Currently, at least 13 RSV-A and 20 RSV-B genotypes have been identified based on the sequencing attachment G gene [6,7,8,9,10,11].

Molecular epidemiology and genetic evolution studies on RSV are crucial for tracking the emergence of new strains and for vaccine development. For example, the RSV-B genotype BA emerged in 1999 with a 60-nucleotide (nt) duplication, and a novel RSV-A genotype, ON1, with a 72-nt duplication in the G gene, was reported in 2010; both have successfully replaced other circulating RSV genotypes and disseminated globally [12,13]. In Taiwan, only one study, from northern Taiwan, investigated the molecular epidemiology of this virus [14]. This study aimed to detect the RSV genotype circulating pattern in central Taiwan between 2008 and 2017 and delineate the genetic variability of Taiwanese RSV strains compared with other RSV strains circulating worldwide.

## 2. Materials and Methods

### 2.1. RSV Isolates

This study obtained RSV isolates from two resources. The first is the clinical virology laboratory at the Taichung Veterans General Hospital (VGH-Taichung), which is one of nine Center for Disease Control contrast virology laboratories in Taiwan. This laboratory is responsible for the respiratory viral isolation of nasopharyngeal samples collected from the hospital and coordinates the satellite surveillance of general clinics in central Taiwan. The presence of seven common respiratory viruses (RSV, influenza A and B, adenovirus, and parainfluenza virus I-III) was screened using indirect immunofluorescent assay (Chemicon, Temecula, CA, USA) and direct fluorescent assay (Diagnostic Hybrids, Athens, OH, USA). All the identified respiratory viruses were routinely stored at −80 °C, as required, and these stored isolates were recoded to remove access to patients’ clinical information. In total, 139 stored RSV isolates from 2008 to 2014 were obtained from the VGH-Taichung laboratory.

In addition, we obtained 74 RSV isolates from the viral etiology study of acute bronchiolitis (*N* = 184) conducted at the Chang Bing Show Chwan Memorial Hospital between 2014 and 2017 (RD103032). This study was approved by the CBSCMH institute review board (No. 1030802).

### 2.2. RNA Extraction, cDNA Synthesis, and G Protein Sequencing

Total viral RNA was extracted using a QIAamp viral RNA minikit (Qiagen, Valencia, CA, USA) according to the manufacturer’s instructions. Reverse transcription was performed using random primers and a Moloney murine leukemia virus reverse transcription kit (Protech). The cDNA was amplified with a previously described nest polymerase chain reaction protocol targeting the G ectodomain region [15], and the amplicons were subjected to Sanger sequencing by using an ABI 3730 automated sequencer (Applied Biosystems). The sequences analyzed in this study have been deposited in GenBank (accession numbers MK344675-MK344719, MK306291-MK306358, MK361044-MK361103, MK387078-MK387080, MK443500, MK460229).

### 2.3. Phylogenetic and Phylodynamic Analysis

A total of 72 RSV G gene sequences (33 RSV-A and 39 RSV-B) were obtained from GenBank for reference. The alignment of the sequences from our study with the reference sequences was performed using the MEGA7 software MUSCLE program. All the RSV-A and RSV-B sequences used in this study were analyzed using a genetic algorithm for recombination detection from the Datamonkey website [16]; no evidence of recombination was revealed. Phylogenetic trees were generated using the neighbor-joining method implemented in MEGA7, with 1000 replicates of bootstrap probabilities for the evaluation of confidence estimates. The Bayesian Markov chain Monte Carlo method was used to determine the evolutionary dynamics of RSV-A and RSV-B in central Taiwan and the nt substitution rate through the BEAST software package. A Bayesian skyline plot (BSP) framework was constructed with a relaxed uncorrected lognormal distribution model through BEAST software to assess the time-course trend of the total circulation of RSV in Taiwan during the investigation period.

### 2.4. Protein Substitution Analysis, Selection Pressure, and Glycosylation Prediction

Deduced amino acid sequences were translated with a standard genetic code by using MEGA7 software. The mutations were described for the RSV ON1 and BA9 strains in this study with respect to their prototype strains: ON67-1210A (GenBank accession number JN257693) and BA4128/99B (GenBank accession number AY333364). Potential positive selected and coevolved sites were estimated through the Datamonkey Web server by using three methods: single likelihood ancestral counting (SLAC), fixed-effects likelihood (FEL), and fast unconstrained Bayesian approximation (FUBAR). The N-glycosylation and O-glycosylation gain and loss of the HVR2 G protein were predicted using the NetNGlyc 1.0 and NetOGlyc 4.0 servers, with a threshold value of 0.5. O-linked glycosylation is based on an aa serine and threonine configuration, and N-linked glycosylation is based on an aa Asn-Xaa-Ser/Thr, except proline configuration.

## 3. Results

### 3.1. Frequency of RSV Isolates and Genotype Distribution in Taiwan, 2008–2017

The 213 RSV isolates used in this study were all successfully sequenced. Of these samples, 132 (62%) were subgrouped as RSV-A and 81 (38%) as RSV-B. Three genotypes were identified as RSV-A: GA2 (1, 0.8%), NA1 (52, 39.4%), and ON1 (79, 59.8%). Four genotypes were identified as RSV-B: GB2 (1, 1.2%), BA9 (76, 93.8%), BA10 (3, 3.7%), and BA12 (1, 1.2%). The annual RSV isolate and genotype distributions are presented in Table 1. The RSV-A and RSV-B viruses cocirculated annually in central Taiwan, with RSV-A predominating in 2008–2012, 2014, and 2015. Regarding the genotype shift over time, the RSV-A NA1 genotype was the predominant type by 2012, and the ON1 genotype has been the dominant type since 2013. For RSV-B, the BA10 genotype appeared in 2008 but was quickly replaced by the BA9 genotype in 2009.

### 3.2. Phylogenetic Analysis of RSV

**Figure 1:**
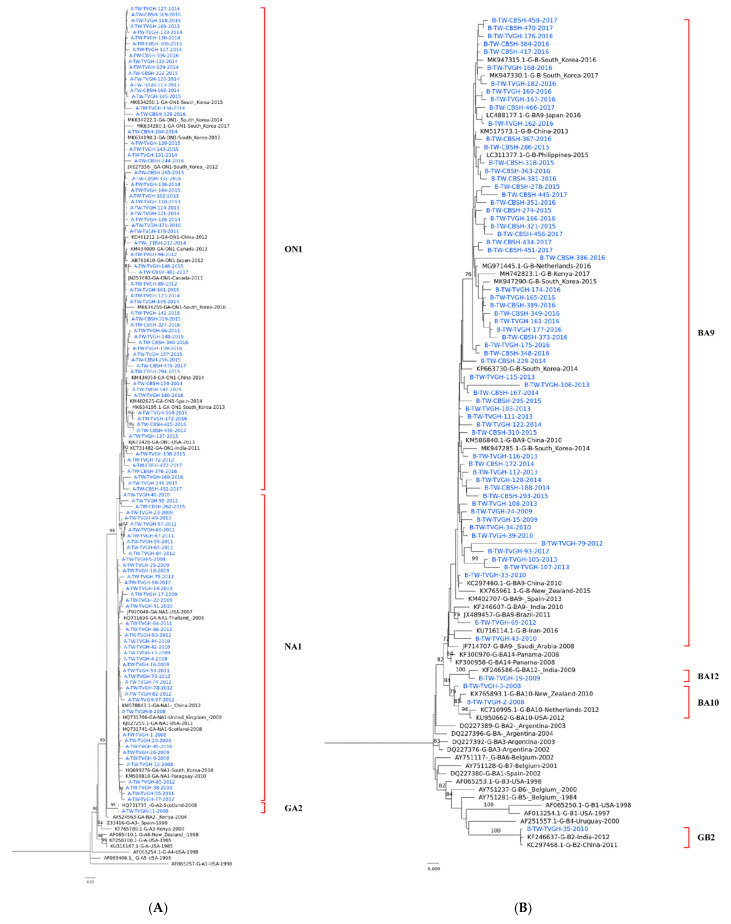
Phylogenetic trees of circulating RSV A and B strains in Taiwan, 2008–2017. Phylogenetic tree of RSV A/RSV B strains and reference sequences of identified genotypes. Phylogenetic trees for RSV A (**A**) and RSV B (**B**) strains were constructed with neighbor-joining method with 1000 bootstrap replicates using MEGA 7 software. The bootstrap value is shown next to the branches (only values ≥70% were shown).

After removal of the identical sequences, a total of 180 sequences, including 114 RSV-A and 66 RSV-B strains of the G protein gene, were retrieved for phylogenetic analysis. The sequences used for the phylogenetic analysis corresponded to 298–897 nt (aa 100–299) and 217–900 nt (aa 91–300), which covered the whole G-ectodomain region. All the RSV-A strains, except one, were clustered into two genotypes: NA1 (45, 39.5%) and ON1(68, 59.6%) (Figure 1A). The Taiwanese NA1 and ON1 strains shared a high bootstrap value with strains from other countries, and the average p-distance within NA1 and ON1 in the cluster was 0.0015 and 0.0016, respectively, with a mean distance between the two being 0.0022.

Among the RSV-B strains, four genotypes were identified: BA9 (62, 93.9%), BA10 (2, 3%), BA12 (1, 1.5%), and GB2 (1, 1.5%) (Figure 1B). BA9 has accounted for the vast majority of investigated strains in Taiwan since 2010, and the average p-distance within BA9 is 0.0029.

### 3.3. Deduced Amino Acid Analysis

**Figure 2:**
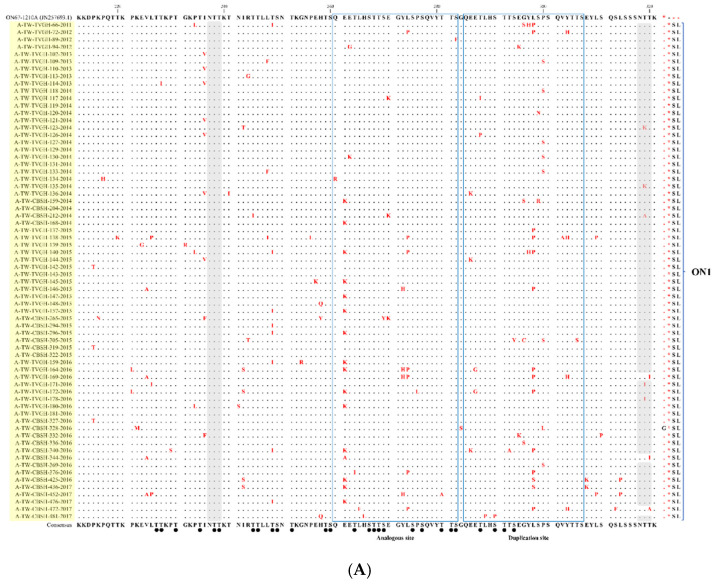

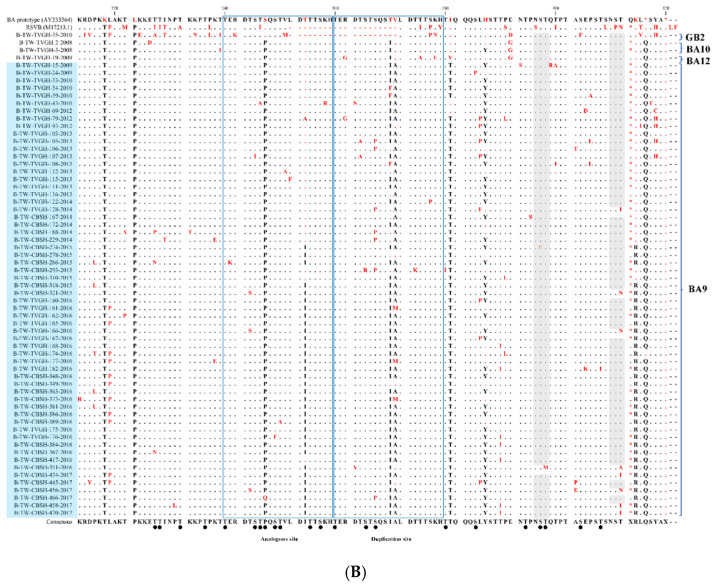
Alignment of the deduced amino acids of the second G protein hypervariable region of the (**A**) ON1 and (**B**) BA9 genotypes in Taiwan. All the representative sequences of the ON1 strains in this study were aligned with the ON67-1210A prototype strain (GenBank accession number: JN257963), and those of the BA9 strains were aligned with the reference strain BA4128/99B (AV333364). The substituted amino acids are indicated in red. The ON1 and BA9 sequences of this study were highlighted by light yellow and light blue colors, respectively. The blue boxed areas indicate analogous and duplicate sites. The predicted N-glycosylation sites are highlighted in gray, and the black dots represent the predicted O-link sites.

Compared with the NA1 prototype (NG-016-04), all the NA1 strains from Taiwan had N260S aa substitution and some had N273Y within the aa sequences. While aligning with the ON1 prototype ON1 (ON67-1210A) (Figure 2A), I236V, I243S, E262K, Y273H, L274P, Y297H, L298P/S, P300S, and Y304H aa substitutions were identified diversely in the Taiwanese ON1 strains. Sites 298 and 300, located inside the duplicated region, and P300S were only identified in strains before 2015, whereas L298P/S was commonly revealed in strains after 2015. Because the sequences of the Taiwan BA strains were aligned with the prototype (AY333364) (Figure 2B), most of these strains exhibited substitutions K218T, L223P, S247P, T270I, V271A, I281T, and H287Y. RSV-B strains carried mutations D253I and K314R, which have appeared since 2015.

Alignment of the G gene HVR2 deduced aa partial sequences of the ON1 and BA9 strains detected in Taiwan between 2008 and 2017 are shown in Figure 2A,B, respectively. A comparison of the reported codon aa changes between this study and other studies is summarized in Supplementary Table S1.

### 3.4. Bayesian Skyline Plot and Evolution Rate

**Figure 3:**
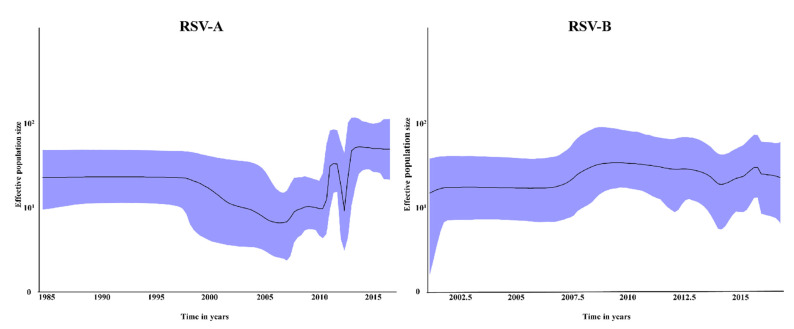
Bayesian skyline plot.

The two BSPs illustrating the time trends for the RSV-A and RSV-B strains circulating in Taiwan are presented in Figure 3. The RSV-B BSP was relatively stable throughout the study period, with the exclusive circulation of the BA9 genotype. By contrast, the RSV-A BSP exhibited an inclined trend after 2008, which fluctuated between 2011 and 2013, corresponding to the emergence of the ON1 genotype in 2011–2013 and the replacement of the original NA1 genotype. Subsequently, the RSV-A BSP presented a steady pattern until the end of the investigation period.

The mean evolutionary rate of the Taiwanese RSV-A and RSV-B strains was estimated at 2.90 × 10^−3^ (95% highest probability density [HPD] interval, 2.26–3.59 × 10^−3^) and 5.23 × 10^−3^ (95% HPD, 4.14–6.37 × 10^−3^) substitutions/site/year, respectively.

Bayesian skyline plot of the Taiwanese RSV-A strain G gene hypervariable region, with the *y*-axis representing the effective population size and the *x*-axis representing the generation time (in years). The solid black line indicates the median effective population size, and the upper and lower limit of the blue area represents the range of 95% HPD.

### 3.5. Selection Pressure Analysis

We analyzed the possible selection pressure on the G gene using three selection models: SLAC, EFL, and FUBAR (Table 2). For the Taiwanese NA1 and ON1 strains, codon 274 was under positive selection pressure, which was consistently identified in all three models. L298P, an ON1 genotype codon, was also under positive selection pressure. For the BA9 genotype, codon 287 was under positive selection pressure, as identified in all three models. However, codon 312 was recognized as a positive selection site only by the FEL and FUBAR models, and codon 270 was identified only by the SLAC model.

### 3.6. N-Linked and O-Linked Glycosylation Site Analysis

For N-glycosylation, most Taiwanese ON1 strains had two predicted sites at aa positions 237 (100%) and 318 (95.6%). T320I substitution was linked to the loss of N-glycosylation. With regard to the NA1 strains, two predicted sites were identified at aa 251 (91.1%) and 294 (60%). Two aa substitutions, N251Y and N294Y, contributed to N-glycosylation loss, whereas D237N substitution led to N-glycosylation gain. The O-glycosylation pattern was genotype specific, and 29–31 predicted sites were identified in the NA1 strains and 21–40 in the ON1 strains.

The majority of the Taiwanese BA9 strains had two predicted N-glycosylation sites at aa positions 296 (98.3%) and 310 (86.7%). The aa substitution T312I/N caused glycosylation loss. O-glycosylation in the BA strains varied between 13 and 40 predicted sites. The detailed loss and gain of the predicted N-glycosylation and O-glycosylation sites in the present study are listed in Table 3 and Supplementary Table S2.

## 4. Discussion

In the present study, we described the molecular epidemiology of the circulating RSV isolates in Taiwan over 10 consecutive years. RSV-A and RSV-B cocirculated in Taiwan, and three main circulating genotypes were identified: NA1, ON1, and BA9. In addition, a circulating period for RSV-A and RSV-B and a shifting of prevailing genotypes were noted, which is consistent with numerous longitudinal studies worldwide [7,9,17,18,19,20]. The NA1 genotype predominated until 2012 and was replaced by genotype ON1, which contained a 72-nt duplication in the C terminal region of the G gene. The genotype shifting from NA1 to ON1, first identified in Canada in 2011, has been widely described as occurring over a 4-year period. In this study, the ON1 genotype was first identified in 2011 and became the solitary prevailing RSV-A genotype in 2013 in Taiwan. This phenomenon was also observed in Ontario, Canada [21], as well as in South Korea, Vietnam, China, Thailand, the Philippines, Madagascar, Italy, and Kenya [7,9,17,19,20,22,23,24].

**Figure 4:**
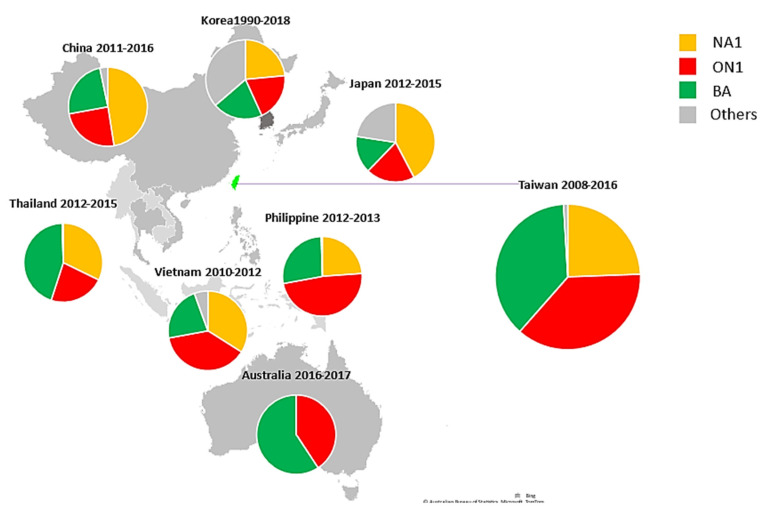
Geographic distribution of the RSV A and RSV B genotypes in the Asia-Pacific region, 2008–2018. (The figure was created with Microsoft PowerPoint and Excel).

With regard to RSV-B, the BA genotype with a 60-nt duplication, originally described in Argentina in 1999, has spread globally and has been categorized into at least 14 subgenotypes [17,25,26]. To date, the BA9 genotype is the prevalent RSV-B globally [19,20,25,27]. The BA9 genotype was first detected separately in Japan and South Korea in the 2005–2006 season and became the dominant genotype in 2009 [10,18]. In our study, BA9 accounted for 93.8% of the RSV-B strains and prevailed throughout the study period except 2011. The RSV genotype distribution in 2008–2018 is summarized by country in the Asia–Pacific region in Figure 4 [9,17,19,22,26], indicating that RSV epidemiology changed temporally but not geographically. The successful spread of the ON1 and BA genotypes globally suggests that the aa insertion in the G gene might facilitate the transmission of new RSV strains by enhancing viral fitness and escaping per-existing population immunity. Our viral sequences in both the RSV-A and RSV-B phylogenetic trees were constantly clustered with reference sequences from other countries in each of the time periods. With the exception of some possible locally circulating strains, this suggests that RSV spreads and circulates globally and has multiple introductions into Taiwan.

The RSV G protein gene has the highest genetic diversity and presents the highest evolutionary rate among the 11 genes of the RSV genome. A phylogenetic analysis using the whole ectodermal region of the G protein is consistent with those using whole genome sequences. In this study, the estimated evolutionary rates of the G protein in RSV-B (5.23 × 10^−3^ substitutions/site/year) were slightly higher than that in RSV-A (2.90 × 10^−3^ substitutions/site/year), which is consistent with reports from the Philippines, South Africa, Senegal, and the United States [9,11,26,28,29] but in contrast to a report from Australia [26]. These variations might be caused by the different data sets and mathematical models used.

RSV accumulates aa changes over time. All the Taiwanese NA1 strains harbored an N260S substitution (reference strain: AB470478.1). L247M, N273Y, and L294P substitutions were identified in some strains and were particularly clustered in strains from 2011. T253I substitution has been reported in the Philippines and Thailand [9,19] but was not observed in this study. Compared with the representative strain (JN257693), I236V, I243S, E262K, Y273H, L274P, Y297H, L298P/S, P300S, and Y304H aa changes were identified in the ON1 strains in our study. Of these, E262K, L274P, L298P, Y304H, and L310P were the five common codon changes shared by the viruses from multiple countries [1,7,8,9,10,17,19,20,24]. E262K and L298P/S substitutions have been identified in Taiwan since 2015, and strains acquiring I243S, E262K, and L298P/S in combination were specifically observed in strains in 2016–17, which was unique to Taiwan (supplementary Figure S1A). However, T245I (the Philippines) [9], G254K (Italy) [7], and L310P (Japan, Germany, Kenya) [8,24,30] were not observed in this study.

For RSV-B, the BA genotype, derived from its ancestor GB5, was characterized by a 60-nt duplication and successfully spread worldwide [27]. When compared with the reference strain AY333364, seven major aa changes were identified in most Taiwanese BA9 strains: K218T, L223P, S247P, I281T, and H287Y (outside the duplication site); T270I and V271A (within the duplication site). These substitution sites have been commonly reported across the world [7,9,10,19,20,25,27]. In addition to the aforementioned sites, a combination of T254I and K314R substitution changes was only seen in strains in 2015–2017. T254I substitution was also reported in RSV strains in China, Korea, Japan, and Australia during the same period (supplementary Figure S1B). However, we were unable to identify a concomitant substitution for K314R in the alignment with sequences from these countries. Instead, the combination of T254I and K314R has been described in the Kenyan RSV BA strains for 2015–2017 [25]. Similar to SARS-CoV2, the RNA virus has a propensity to mutate over time, leading to the emergence of highly transmissible viral variants [31]. The acquisition of T254I and K314R substitutions might be an explanation for the shifting predominance of the BA9 genotype in the 2016–2017 epidemic seasons in Taiwan.

A virus can also escape host immunity by altering protein glycosylation. For RSV, proteins G and F are the main antigenic sites. The glycosylation pattern of the F protein is relatively conserved [29]. The number of putative O-linked glycosylation sites for the RSV G protein varies across studies, whereas the N-linked glycosylation sequons in the G gene tend to be genotype specific [25,29]. In the HVR2 region, the putative N-glycosylation sites for the NA1 genotype included N237, N251, and N294. N237D/Y aa substitution (reference to A2_M74586) was common in the present study as well as in the reports from Vietnam, the Philippines, Thailand, China, and Madagascar [9,19,20,22,23], involving N-linked glycosylation loss. The N-linked glycosylation sites of the BA9 genotype were typically conserved at sites 296 and 310 [9,19,20,27], but the aa change in T312N/I in our study contributed to a glycosylation loss. All the predicted N-glycosylation sites in N237 and N318 have been highly conserved for the ON1 strains in most published studies [9,20,24,30].

At least four aa codons of the ON1 genotype have been reported under positive selection pressure: aa 225, 226, 274, and 290. In our study, aa 274 and 298 were under positive selection pressure according to all three selection models. For the Taiwanese NA1 strains, only L274P was identified as significant. These findings are consistent with previous studies [9,19,21,24]. Aa 237 and 274 have been linked to viral immune escape and close proximity to the predicted glycosylation motifs [5]. D237A and L274P in RSV-A are known to cause N-glycosylation loss. Among the BA9 strains, three substitutions (position 270, 287, and 312) were proposed as being under positive selection pressure, but none of these was confirmed by ≥2 models. Site 270 is located in the 60-nt duplication region of the RSV-B strains. Thirteen different positively selected aas have been identified in the global BA9 lineages, and six of them have been demonstrated to be highly selected (219, 267, 270, 287, 297, and 305) [27].

One major limitation in our study is that the clinical data are not available for the 2008–2015 samples. Therefore, we cannot assess the relationship between RSV genotypes or specific mutations and clinical severity.

In conclusion, this study explored RSV epidemiology, genotype shifting, and variability in central Taiwan from 2008 to 2017. Genotypes NA1, ON1, and BA9 were the predominant circulating strains over 10 consecutive seasons, continuously evolving over time. Continuing local and global surveillance is key to understanding RSV epidemiology and evolution, which is necessary for vaccine development and prevention strategies.

## Figures and Tables

**Table 1:** Annual distribution of 213 RSV isolates in Taiwan, 2008–2017.

Year	2008	2009	2010	2011	2012	2013	2014	2015	2016	2017
**Total**	10	13	15	11	21	15	29	32	52	15
**RSV A**										
**GA2**	1 (10)	0	0	0	0	0	0	0	0	0
**NA1**	6 (60)	10 (77)	9 (60)	10 (91)	15 (71)	0	0	2 (6)	0	0
**ON1**	0	0	0	1 (9)	3 (14)	6 (40)	22 (76)	21 (66)	20 (38)	6 (40)
**RSV B**										
**GB2**	0	0	1 (7)	0	0	0	0	0	0	0
**BA9**	1 (10)	2 (15)	4 (27)	0	3 (14)	9 (60)	7 (24)	9 (28)	32 (62)	9 (60)
**BA10**	2 (20)	0	1 (7)	0	0	0	0	0	0	0
**BA12**	0	1 (8)	0	0	0	0	0	0	0	0

Data in cells were presented as *n* (%).

**Table 2:** Putative sites under positive selection in RSV strains analyzed in Taiwan.

Genotype	SLAC		FEL		FUBAR	
	dN/dS Mean	Amino Acid Substitution *	dN/dS Mean	Amino Acid Substitution	BayesFactor	Amino Acid Substitution
NA1	0.657	L274P	0.647	L274P **	25.152	L274P
ON1	0.625	L274P	0.616	L274P	27.933	L274P
		L298P		L298P	57.545	L298P
BA9	0.468	H287Y **	0.455	H287Y	79.643	H287Y
		T270I ** T270F **		T312N T312A T312I	97.297	T312N T312A T312I

Amino acid substitutions under positive selection pressure were examined using conservative single likelihood ancestor counting (SLAC), fixed effects likelihood (FEL), and fast unconstrained Bayesian approximation (FUBAR) models. FUBAR results were assessed posterior probability of 0.95. * The amino acid substitution selection site *p*-value cutoff of 0.05. ** non-significance.

**Table 3:** Putative N-glycosylation sites of the RSV genotypes NA1, ON1, and BA9 in Taiwan.

Genotype	Putative N-Glycosylation Site % (*n*/N)		
NA1	N266	2.2%	(1/45)
	N237	4.4%	(2/45)
	N251	91.1%	(41/45)
	N294	60%	(27/45)
ON1	N237	100.0%	(68/68)
	N318	95.6%	(65/68)
	N242	1.5%	(1/68)
BA9	N296	98.3%	(59/60)
	N310	86.7%	(52/60)

## Data Availability

Not applicable.

## Supplementary Materials

The following supporting information can be downloaded at: https://www.mdpi.com/article/10.3390/v14010032/s1, Figure S1: (**A**) Alignment of deduced amino acid from Taiwan ON1 strains with representative strains from other countries, (**B**) Alignment of deduced amino acid from Taiwan BA9 strains with representative strains from other countries; Table S1: Reported codon changes of 2ND HVR in G gene among circulating ON1 and BA9 genotype by countries; Table S2: Specimen with G protein substitution and their predicted effect on glycosylation: NA1, ON1 and BA9 genotype.
