# Supplementary material for: Genetic Diversity and Molecular Epidemiology of Circulating Respiratory Syncytial Virus in Central Taiwan, 2008–2017
Source: Viruses. 2021 Dec 24;14(1):32. doi: 10.3390/v14010032 (PMC8777914; doi:10.3390/v14010032)
Supplement: Supplementary file 1 [file viruses-14-00032-s001.zip › viruses-1454513- corrected-done.pdf]

viruses

Article

Genetic Diversity and Molecular Epidemiology of Circulating Respiratory Syncytial Virus in Central Taiwan, 2008–2017

Chun Yi Lee [1,2], Yu Ping Fang [1], Li Chung Wang [3], Teh Ying Chou [2,4], and Hsin Fu Liu [5,6,7,*]

1 Department of Pediatrics, Chang Bing Show Chwan Memorial Hospital, Changhua 505029, Taiwan; lee821083@gmail.com (C.Y.L.); y20050401@gmail.com (Y.P.F.)
2 Institute of Clinical Medicine, National Yang Ming Chiao Tung University, Taipei 112304, Taiwan; tychou@vghtpe.gov.tw
3 Department of Pathology and Laboratory Medicine, Taichung Veterans General Hospital, Taichung 40705, Taiwan; lcwang@vghtc.gov.tw
4 Department of Pathology and Laboratory Medicine, Taipei Veterans General Hospital, Taipei 11217, Taiwan
5 Department of Medical Research, Mackay Memorial Hospital, Taipei 25160, Taiwan
6 Institute of Bioscience and Biotechnology, National Taiwan Ocean University, Keelung, 202301, Taiwan
7 Institute of Biomedical Sciences, MacKay Medical College, New Taipei City 25245, Taiwan
* Correspondence: hsinfu@mmh.org.tw

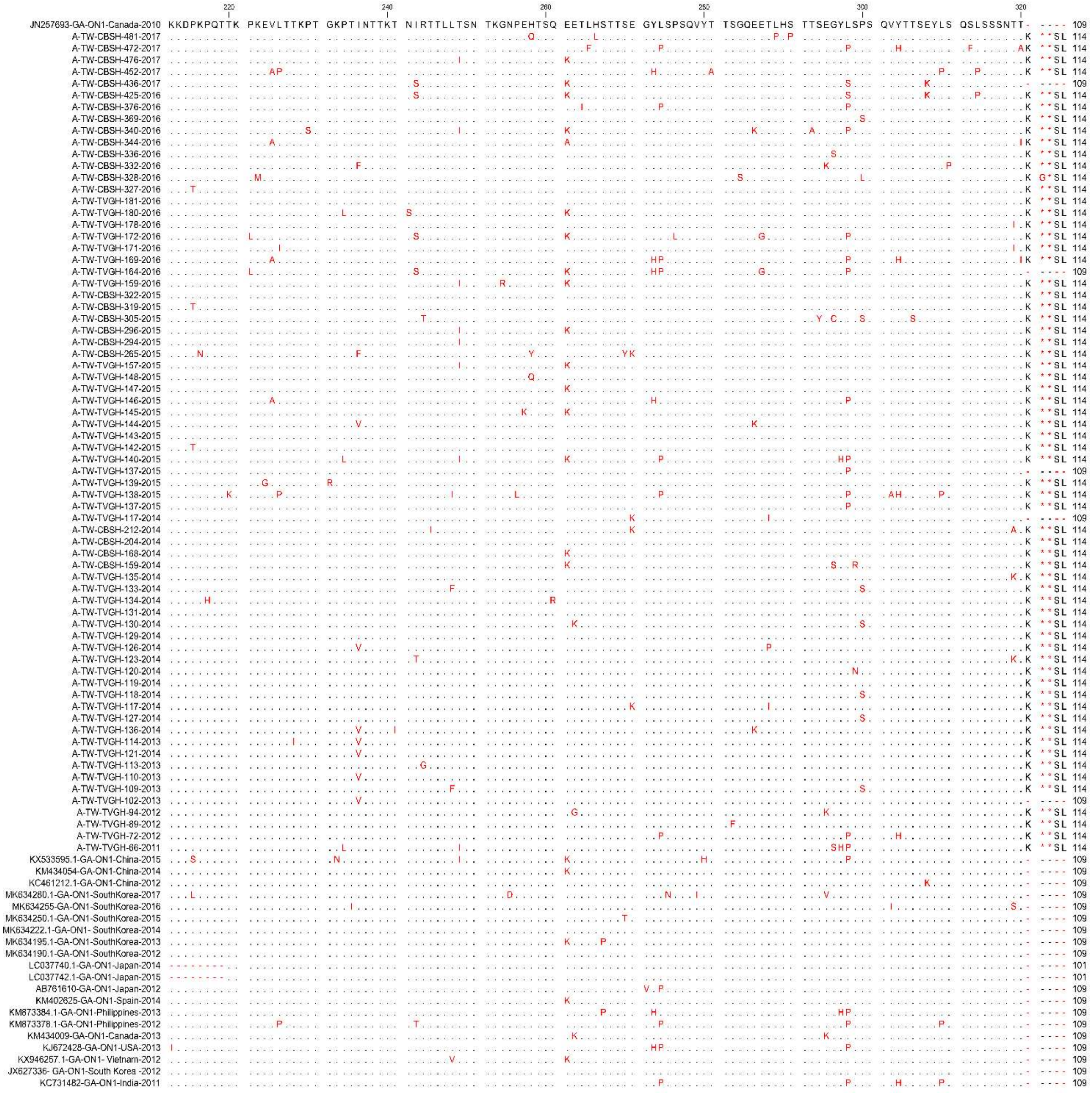

(**A**)

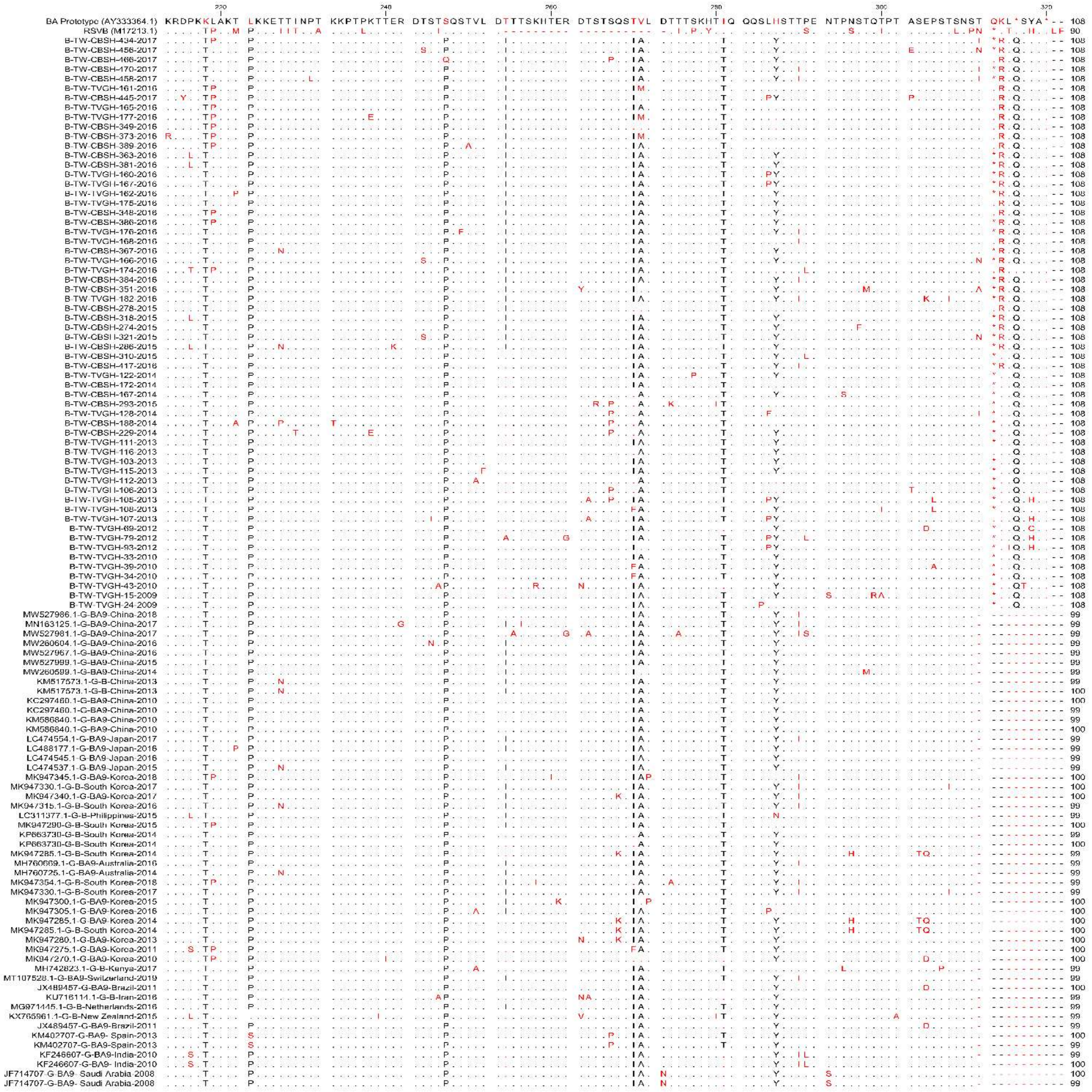

(**B**)

Figure S1. (**A**) Alignment of deduced amino acid from Taiwan ON1 strains with representative strains from other countries. (**B**) Alignment of deduced amino acid from Taiwan BA9 strains with representative strains from other countries.

Table S1. Reported codon changes of 2ND HVR in G gene among circulating ON1 and BA9 genotype by countries. (A) ON1 genotype. (B) BA 9 genotype.

(A)

ON1	236	243	245	258	262	270	271	274	298	300	304	306	308	310
Taiwan (2008-2017)	I/V	I/T			E/K			L/P	L/P	P/S	Y/H			L/P
Germany (2012-2013)								L/P	L/P		Y/H		E/K	
Korea (1990-2018)					E/K			L/P	L/P		Y/H			
Philippine (2012-2013)			T/I	H/Q				L/P	L/P					L/P
Thailand (2012-2015)					E/K		E/K		L/P		Y/H			
Vietnam (2010-2012)					E/K			L/P	L/P		Y/H			L/P
Kenya (2010-2015)								L/P	L/P		Y/H			L/P
Madagascar (2011-2017)		I/S			E/K				L/P					
Netherlands (2016-2017)		I/T/S			E/K	S/Y	E/K	L/P	L/P	P/S	Y/H	T/I		L/P
Italy (2009-2014)					E/K			L/P	L/P		Y/H			L/P

(B)

	218	219	223	247	254	267	270	271	281	287	314
Taiwan (2008-2017)	K/T	L/P	L/P	S/P	T/I	S/P	T/I	V/A	I/T	H/Y	K/R
Germany (2012-2013)	K/T		L/P	S/P			T/I	V/A	I/T	H/Y	
Korea (1990-2018)				S/P	T/I		T/I	V/A			
Philippine (2012-2013)	K/T		L/P	S/P		S/P	T/I	V/A			
Vietnam (2010-2012)			L/P	S/P				V/A		H/Y	
Kenya (2003-2017)					T/I	S/P	T/I	V/A			K/R
Madagascar (2011-2017)	K/T		L/P	S/P		S/P	T/I	V/A	I/T	H/Y	
China (2011-2016)	K/T		L/P	S/P			T/I	V/A	I/T	H/Y	
Thailand (2012-2015)	K/T		L/P	S/P	T/I		T/I	V/A	I/T	H/Y	
Italy (2009-2014)	K/T		L/P	S/P			T/I	V/A	I/T	H/Y	

Deduced amino acid change was reference to prototype strain: ON1 (JN257693) and BA (AY333364). Cells shaded by yellow-brown color mean codon changes noted sporadically; Cells highlighted by pink indicated these codon changes were found in particular study years.

Table S2. Specimen with G protein substitution and their predicted effect on glycosylation: NA1, ON1 and BA9 genotype.

Genotype and strain ID	Substitution	Effect on glycosylation	Number of O-glycosylation	Number of N-glycosylation
RSVA-NA1				
A/TW/TVGH-38/2010	N251Y<br>N294Y	-N-Glu	30	0
A/TW/TVGH-55/2011	N251Y<br>N294Y	-N-Glu	29	0
A/TW/TVGH-77/2012	N251Y<br>N294Y	-N-Glu	29	0
A/TW/TVGH-85/2012	N251Y	-N-Glu	30	1
A/TW/TVGH-40/2010	D237N	+N-Glu	30	3
A/TW/TVGH-95/2012	D237N	+N-Glu	31	3
A/TW/TVGH-78/2012	H266N	-	30	2
RSVA-ON1				
A/TW/TVGH-169/2016	T320I	-N-Glu	32	1
A/TW/CBSH-344/2016	T320I	-N-Glu	27	1
A/TW/CBSH-472/2017	T320A	-N-Glu	29	1
A/TW/CBSH-340/2016	P230S	+O-Glu	35	2
A/TW/TVGH-180/2016	N242S	+O-Glu	28	2
A/TW/TVGH-164/2016	I243S	+O-Glu	31	2
A/TW/TVGH-172/2016	I243S	+O-Glu	40	2
A/TW/CBSH-425/2016	I243S<br>L298S	+O-Glu	37	2
A/TW/CBSH-436/2016	I243S<br>L298S	+O-Glu	33	2
A/TW/CBSH-328/2016	G284S	+O-Glu	25	2
A/TW/TVGH-66/2012	G296S	+O-Glu	36	2
A/TW/TVGH-123/2014	I243S	+O-Glu	28	2
A/TW/TVGH-114/2013	T238I	-O-Glu	28	2
A/TW/CBSH-212/2014	T2445I	-O-Glu	31	2
A/TW/TVGH-66/2011	T249I	-O-Glu	36	2
A/TW/CBSH-294/2015	T249I	-O-Glu	22	2
A/TW/TVGH-157/2015	T249I	-O-Glu	25	2
A/TW/CBSH-296/2015	T249I	-O-Glu	25	2
A/TW/TVGH-140/2015	T249I	-O-Glu	28	2
A/TW/TVGH-159/2016	T249I	-O-Glu	23	2
A/TW/CBSH-340/2016	T249I	-O-Glu	35	2
A/TW/CBSH-376/2016	T264I	-O-Glu	31	2
A/TW/CBSH-265/2015	S270Y	-O-Glu	36	2
A/TW/CBSH-452/2017	T281A	-O-Glu	38	2
A/TW/TVGH-89/2012	S283F	-O-Glu	21	2

A/TW/TVGH-117/2014	T288I	-O-Glu	25	2
A/TW/TVGH-126/2014	T288P	-O-Glu	27	2
A/TW/CBSH-305/2015	S294Y	-O-Glu	22	2
B/TW/TVGH-128/2014	T312I	-N-Glu	23	1
B/TW/CBSH-321/2015	T312N	-N-Glu	18	1
B/TW/TVGH-166/2016	T312N	-N-Glu	18	1
B/TW/CBSH-351/2016	T298M<br>T312A	-N-Glu	13	0
B/TW/CBSH-434/2017	T312I	-N-Glu	16	1
B/TW/CBSH-456/2017	T312N	-N-Glu	17	1
B/TW/CBSH-458/2017	T312I	-N-Glu	14	1
B/TW/CBSH-470/2017	T31I	-N-Glu	14	1
B/TW/CBSH-188/2014	T227P<br>K233T	-O-Glu<br>+O-Glu	24	2
B/TW/CBSH-286/2015	T227N<br>T240K	-O-Glu<br>-O-Glu	19	2
B/TW/CBSH-367/2016	T227N	-O-Glu	15	2
B/TW/CBSH-321/2015	T244S	-	18	1
B/TW/TVSH-166/2016	T244S	-	18	1
B/TW/CBSH-456/2017	T244S	-	17	1
B/TW/TVGH-107/2013	S245I	-O-Glu	20	2
B/TW/TVGH-43/2010	T246A	-O-Glu	17	2
B/TW/TVGH-176/2016	S249F	-O-Glu	13	2
B/TW/CBSH-389/2016	T250A	-O-Glu	16	2
B/TW/CBSH-293/2015	S265R<br>T280I	-O-Glu<br>-O-Glu	19	2
B/TW/TVGH-24/2009	S285P	-O-Glu	26	2
B/TW/CBSH-274/2015	S297F	-O-Glu	15	2
B/TW/CBSH-351/2016	T298M	-O-Glu	13	0
B/TW/TVGH-182/2016	T308I	-O-Glu	14	2
